# The Enigma of Lymphocyte Apoptosis in the Response to Influenza Virus Infection

**DOI:** 10.3390/v15030759

**Published:** 2023-03-16

**Authors:** Norbert J. Roberts

**Affiliations:** 1 Division of Infectious Diseases and Immunology, Department of Medicine, New York University Grossman School of Medicine, New York, NY 10016, USA; norbert.roberts@nyulangone.org; 2 Division of Infectious Diseases, Department of Internal Medicine, University of Texas Medical Branch, Gaveston, TX 77555, USA

**Keywords:** influenza virus, human monocytes, human macrophages, human lymphocytes, lymphocyte apoptosis

## Abstract

In the pathogenesis of influenza virus infection, lymphocyte apoptosis as a part of the infection and/or the immune response to the virus can be somewhat puzzling. The percentage of human T lymphocytes within the peripheral blood mononuclear cell population that becomes apoptotic greatly exceeds the percentage that are infected after exposure to the virus, consistent with substantial apoptosis of bystander T lymphocytes. Studies reveal an important role of viral neuraminidase expression by co-cultured monocyte/macrophages in induction of apoptosis, including that of uninfected bystander lymphocytes. Despite these observations, it is a reasonable perspective to recognize that the development of lymphocyte apoptosis during the response to infection does not preclude a successful immune response and recovery of the infected host in the great majority of cases. Further investigation is clearly warranted to understand its role in the pathogenesis of influenza virus infection for human subjects.

## 1. Introduction

Influenza A virus (IAV) is a major cause of respiratory infections in the United States and worldwide, with the most severe cases occurring in the very young and in elderly individuals [1,2,3]. Although the highest illness rates occur in children, most deaths occur with infections of the elderly [2,4,5]. In addition, immunocompromised individuals of any age are at greater risk of adverse outcomes [6]. In contrast to those observations regarding seasonal IAV infections, pandemic IAV infections, including the most recent and relatively mild H1N1pdm2009, result in an increase in mortality of younger adults [5,7,8].

There have been four influenza pandemics in the last 103 years, in 1918, 1957, 1968 and 2009. Viral strains with pandemic potential continue to be isolated from avian species [8,9]. Avian IAV that have been transmitted to humans include the extensively documented H5N1 and H7N9 viruses as well as H7N7, H9N2, H7N3 and H10N7 viruses [10].

Circulating lymphopenia is a clinical feature of influenza infections caused by seasonal influenza [4,11], avian H5N1 [12], and A(H1N1) pdm09 viruses [13], as also noted by Valero-Pacheco et al. [14] and others [15]. Lymphopenia has been associated with the severity of respiratory viral infection [16]. Several non-exclusive mechanisms for the development of circulating lymphopenia during IAV infection have been postulated, including: direct toxic effect of the virus infection of the cells, although a small percentage (1.4–7%) of lymphocytes in the peripheral blood mononuclear cells (PBMC) are evidently infected [17,18]; re-distribution of lymphocytes to the challenged respiratory tract, with lymphocyte sequestration in the lungs considered a potential major pathway for the depletion of blood lymphocytes [4,16,19]; susceptibility of the infected cells (including lymphocytes) to the developing immune response to the challenge, such as cytotoxic T lymphocyte (CTL)-mediated killing, or “fratricide” [6], as well as preferential targeting of activated lymphocytes that is actually antigen-independent but can suppress the adaptive immune response to IAV [15]; and virus-induced apoptosis of the cells, demonstrated for 15–40% of the lymphocytes depending in part upon the time after exposure [18,20,21]. CD8+ CTL contribute to IAV clearance by direct lysis of the infected cells or by expression of death receptor ligands, with both mechanisms resulting in apoptosis of the infected cells [22]. IAV can induce apoptosis of several cell types, including peripheral blood-derived macrophages [23], avian cell lines [24], NK cells [25] and T lymphocytes from healthy subjects [26]. Lungs of patients infected by highly pathogenic avian influenza (HPAI) virus, such as the H5N1 virus, as well as patients with lethal human IAV infection, contain infiltrating apoptotic leukocytes [27]. Apoptosis of uninfected bystander cells has been considered an important mechanism for lymphocyte depletion induced by viral infections in general [16,18]. In patients who succumb to IAV infection, lung autopsies al-most always show diffuse damage but identify viral RNA in only a subset of patients [28].

A greater understanding of the pathogenesis of IAV infection is needed, including events in the leukocyte response to the challenge that are to a degree puzzling, such as lymphocyte apoptosis as a part of the infection and/or the immune response to the virus. A perspective regarding the existence and extent of lymphocyte apoptosis, especially that of uninfected bystander cells, is offered for consideration.

## 2. Lymphocyte Apoptosis upon Challenge of Human Leukocytes by IAV

Studies have been undertaken in our laboratories to delineate the extent and complexity, and analyze possible mechanisms, behind one of the potentially major contributors to IAV-induced leukopenia, namely the induction of apoptosis of human lymphocytes by the virus. Analysis of lymphocyte subpopulations after exposure of PBMC to IAV showed that a portion of CD3+, CD4+, CD8+, and CD19+ T lymphocytes became apoptotic, measured using a TUNEL assay (terminal deoxynucleotidyltransferase-mediated dUTP-biotin nick end labeling-positive) [18]. In these initial experiments, TUNEL assay results were confirmed using classic tests for DNA fragmentation. A significantly higher percentage of CD8+ T lymphocytes exhibited apoptosis after exposure to the virus than did CD4+ T lymphocytes in the PBMC. The percentage of lymphocytes that were infected was shown to be less than the percentage of apoptotic cells, suggesting that direct effects of cell infection by the virus could not account fully for the high level of cell death. Removal of monocytes/macrophages after exposure to IAV reduced the percent of lymphocytes that were apoptotic. Treatment of virus exposed cultures with anti-TNF (tumor necrosis factor-α) antibody did not reduce the percentage of lymphocytes that were apoptotic. In virus exposed cultures treated with anti-FasL antibody, recombinant soluble human Fas, Ac-DEVD-CHO (caspase-3 inhibitor), or Z-VAD-FMK (general caspase inhibitor), production of the active form of caspase-3 and apoptosis were reduced. The apoptotic cells were Fas-high-density cells while the nonapoptotic cells expressed a low density of Fas. The studies showed that Fas-FasL signaling plays a major role in the induction of apoptosis in human lymphocytes after exposure to IAV. It was suggested that, since the host response to IAV commonly results in recovery from the infection with residual disease uncommon, lymphocyte apoptosis likely represents a part of an overall beneficial immune response as well as a possible mechanism of disease pathogenesis.

Further studies confirmed that the percentage of cells that are infected by IAV is less than the percent of apoptotic cells after exposure [20]. Depletion of monocytes/macrophages and depletion of cells expressing influenza neuraminidase from the cultures after exposure to the virus decreased lymphocyte apoptosis. Treatment of virus-exposed leukocyte cultures with anti-neuraminidase antibodies, but not with anti-hemagglutinin antibodies, reduced lymphocyte production of active caspase-3 and induction of apoptosis. Furthermore, different strains of virus induced different levels of apoptosis and the variations in induction of apoptosis correlated with production and expression of viral neuraminidase by infected leukocytes. The results suggested that cell surface expression of neuraminidase plays an important role in the induction of apoptosis in human lymphocytes by IAV.

Additional studies showed that, whereas exposure of PBMC to infectious IAV induced lymphocyte apoptosis, exposure to inactivated IAV did not result in apoptosis beyond that of sham-exposed cells [21]. This in turn suggests that active synthesis and expression of the neuraminidase by abortively infected monocytes/macrophages is required to induce the death of the lymphocytes. The experiments overall indicated a possible mechanism for the observation that the percent of apoptotic lymphocytes after exposure to IAV greatly exceeds the percent of infected lymphocytes and identified one important risk to bystander cells in the PBMC population.

Studies by other investigators indicate that interferons (IFN) can greatly sensitize certain tissue culture cells such as murine fibroblasts, to apoptosis in response to IAV [29]. IFNs may limit IAV replication by shutting down protein synthesis and triggering apoptosis [30] by inducing multiple components of the death receptor family, including in naïve T lymphocytes [29]. CTLs can induce apoptosis by expressing cytokines such as TNF, FasL, and TNF-related apoptosis-inducing ligand (TRAIL) which recruit death receptors in IAV-infected cells [31,32].

Studies of in vivo human IAV-induced apoptosis are apparently limited to postmortem examinations in cases of severe lethal infection. As noted above, lungs of patients infected by highly pathogenic avian influenza (HPAI) virus, such as the H5N1 virus, as well as patients with lethal human IAV infection, contain infiltrating apoptotic leukocytes [27].

## 3. Is Apoptosis in Response to IAV Defensive or Deleterious?

IAV infection-induced apoptosis of lymphocytes can be considered an important aspect of IAV pathogenesis since lymphocytes play critical roles in defense against and recovery from IAV infections. Divergent concepts regarding the ultimate role(s) of apoptosis during IAV infection have been raised mainly by studies examining cells other than leukocytes and using non-human models such as mice [31]. This may in part be due to the fact that IAV infection of human monocytes/macrophages and lymphocytes is abortive, with evidence of virus-directed protein synthesis, but without the release of free, infectious viral progeny [6,17,33,34,35]. The consequences of IAV-induced cell death would depend in a large part on the cells affected, such as respiratory epithelial cells or monocyte/macrophages and lymphocytes [36].

Studies of pro-apoptotic and anti-apoptotic members of the cellular Bcl-2 protein family have been reported to show that the cell and IAV struggle to control apoptosis, with appropriately timed apoptosis of Madin–Darby canine kidney (MDCK) cells and mouse fibroblasts being important for the replication of IAV [37]. Wurzer et al. reported that caspase 3 activation is essential for efficient IAV propagation in cell lines including MDCK, Vero, and the human epithelial cell line A549 [38]. Interfering with the expression or the function of this major virus induced apoptosis effector using small interfering RNAs impaired propagation in the cells, and replication could be boosted in cells deficient for caspase 3 by ectopic expression of the protein. The avian H5N1 virus NS1 protein has been shown to induce apoptosis in human airway epithelial cells, mainly through a caspase-dependent pathway [39]. Lethal H5N1 infection in mice has been associated with apoptosis of IAV-specific CD8+ T cells in the lung draining lymph nodes [40,41], which might be expected to affect antiviral defense adversely.

On the other hand, IAV-infected mouse and fruit fly cells, the latter host lacking adaptive immunity, were shown to undergo apoptosis and subsequent engulfment by macrophages and neutrophils, representing an apparent antiviral innate immune response mechanism that is conserved among multicellular organisms, according to Nainu et al. [42]. Highly pathogenic avian H5N1 IAV has been reported to delay apoptotic responses in human primary bronchial and alveolar epithelial cells compared to low pathogenic human H1N1 IAV [43], prolonging the duration of viral replication and allowing development of a damaging immune response.

Thus, death receptor-mediated apoptosis represents a complex mechanism used by both the virus and the host to differing degrees depending upon the identity of the challenging virus [21,44]. The infected host cell may use it as part of the antiviral response, to eliminate cells that may be producing viral progeny, whereas some viruses appear to balance apoptotic and anti-apoptotic processes to facilitate their infection of cells as well as their dissemination.

An alternative approach to the treatment of viral diseases has been proposed by some authors, namely, therapeutic strategies to enhance death receptor-mediated apoptotic clearance of virus-infected cells [45]. Such an approach may be beneficial in some viral infections but, in contrast, enhancement of death receptor-mediated events may be deleterious in viral infections in which pathogenesis and propagation are in turn enhanced by apoptosis. Whether such an approach would be likely to benefit the IAV-infected host is unclear currently. Thus, the benefit, or cost, to the host of apoptosis associated with IAV infection warrants continued investigation [31].

## 4. Is lymphocyte Apoptosis in Response to IAV Representative of Responses to Other Viruses?

Perhaps the clearest indication that exposure to another RNA respiratory virus may not exhibit the lymphocyte apoptosis induced by IAV is provided by concomitant analyses of autologous human PBMC exposed to IAV or to respiratory syncytial virus (RSV) [21]. The PBMC had similar percentages of lymphocytes specific for IAV and RSV, and exposure to IAV induced apoptosis as expected. However, exposure to RSV induced far less apoptosis of CD3+ T lymphocytes than did exposure to IAV. Exposure to inactivated IAV or RSV in the same series of experiments did not induce apoptosis relative to sham-exposed PBMC.

Studies of other viruses can be cited relevant to the role of monocytes/macrophages in induction of lymphocyte apoptosis after exposure to a virus as described above. Thus, for example, macrophages infected by bovine viral diarrhea virus have been reported to induce lymphocyte apoptosis [46], and an African swine fever virus-derived protein, from infected macrophages, induced PBMC and macrophage apoptosis [47]. Apoptosis of bystander uninfected CD4+ T lymphocytes by neighboring infected cells has been observed in cell culture and lymphoid tissue of HIV-infected individuals [48]. Furthermore, the number of HIV-infected cells in patients is relatively low and cannot fully account for the loss of CD4+ T lymphocytes in vivo [49]. The loss is thus considered to be due in a large part to the induction of apoptosis in bystander cells, with the HIV envelope glycoprotein designated as a major inducer of cell death [49]. The HIV-induced macrophage-dependent killing was reported to target CD4+ but not CD8+ T lymphocytes, and direct contact between macrophages and lymphocytes was required [48]. Study results supported a role for macrophage-associated FasL and TNF in the depletion of lymphocytes. This process is not unlike that described above for influenza virus infection and expression of its neuraminidase by infected cells. Caprine arthritis encephalitis virus (CAEV), with tropism for macrophages and inability to infect lymphocytes, was modified to express SIV Nef, which did not affect those characteristics [50]. The CAEV-Nef-infected macrophages induced activation and then apoptosis of bystander co-cultured lymphocytes.

It is very interesting and even provocative that monocytes/macrophages, long considered to have a central role in antiviral defense, such as by production of antiviral factors such as interferon [51] and by serving as antigen-presenting cells for activation of lymphocytes and development of the adaptive immune response [52,53], are also required for human IAV infection of lymphocytes [17,54] and for development of lymphocyte apoptosis [18,20], the latter reviewed briefly above. In the airway and lung, monocytes/macrophages establish direct contact with IAV-infected epithelial cells and induce epithelial cell apoptosis [55].

## 5. Influenza Infection and Apoptosis in the Special Environment of the Alveoli

Respiratory epithelial cells, including those of the alveoli, are the desired IAV target and are supportive of IAV replication [56,57]. Features of the alveoli that may influence the development of lymphocyte apoptosis during IAV infection have been described extensively, independent of consideration of apoptosis. Notably, the susceptibility of human mononuclear phagocytes to IAV varies dramatically. Studies indicate that both human monocytes and macrophages are susceptible to IAV infection but may become more susceptible as the monocytes mature into macrophages [58,59]. However, fully differentiated alveolar macrophages have been shown not to be susceptible to direct infection by human IAV [60,61] although they can be infected by highly pathogenic avian strains (HPAI) of IAV such as the H5N1 virus [62,63]. Variation in infectivity of alveolar macrophages described in early studies depended on histological examination for viral antigens in those phagocytic cells; however, recruited peripheral blood monocytes/macrophages, in their mature macrophage form, may resemble AMs histologically [64].

Opportunities do exist for monocyte/macrophage expression of IAV neuraminidase and induction of lymphocyte apoptosis as described in Section 2 above, in the alveoli. Human alveolar macrophages can be infected indirectly by IAV and synthesize and express neuraminidase by ingestion of IAV-specific antibody coated virions [60], with individuals having such virus-specific antibodies if the challenging virus strain is not substantially drifted or shifted from past natural exposure or vaccination. Furthermore, murine models of IAV infection have demonstrated a substantial recruitment of monocytes/macrophages as well as lymphocytes to the lung after IAV challenge [65,66]. Thus, in the setting of IAV challenge, induction of lymphocyte apoptosis through contact with infected monocyte/macrophages is possible and even likely. There are no published data regarding whether IAV neuraminidase expression by infected alveolar epithelial cells would induce lymphocyte apoptosis.

Apoptosis of IAV-infected cells makes them susceptible to phagocytosis [67], and this mechanism for elimination of the virus is conserved among multicellular organisms [42]. Such a defense could be expected to be of greatest benefit if the infected apoptotic cells would have supported a productive infection by the virus, such as respiratory epithelial cells, and perhaps less so in the case of human PBMC for which both monocytes/macrophages and lymphocytes demonstrate abortive infection [17].

## 6. Concluding Remarks with a Perspective Regarding Human Defenses against IAV Infection

Influenza A virus infections are a continued threat to humans [8] and addressing the unknowns in the pathogenesis of the infection continues to be warranted. The potential for a protective role for the host of bystander lymphocyte apoptosis is presently unclear. As noted above, observations regarding IAV-induced lymphocyte apoptosis in survivors of seasonal or pandemic infections are apparently not available. However, relevant studies regarding the role of apoptosis (in general) in the human response to IAV vaccine have been reported. The authors studied young and elderly individuals to determine immune parameters that might predict responsiveness to inactivated IAV vaccine administration and identified nine variables that predicted the antibody response with a high degree of accuracy [68]. Three apoptosis-related variables correlated positively with a strong vaccine response. The authors then studied the response to inactivated IAV vaccine in mice lacking Fas and showed a significant reduction in their ability to produce IAV-specific antibodies, suggesting an important role for apoptosis in the antibody response in vivo. These observations suggest that apoptosis in the setting of IAV challenge may be part of an effective host defense.

In examining a process such as human lymphocyte apoptosis in the setting of IAV infection, it is reasonable and important to consider a teleological argument regarding the leukocyte responses. The vast majority of individuals survive the infection and are endowed with a substantial degree of resistance to re-challenge. They are usually infected again with a virus that has drifted or shifted away from their ability to recognize it immunologically and, even then, individuals are likely to survive the recurring challenge. The overall response to IAV supports survival of the human species. In the end, it is possible that lymphocyte cell death through apoptosis is part of an ultimately effective defense against the virus rather than an adverse event during infection that the host can survive. It remains to be determined what benefit is provided the host or the virus by the substantial bystander lymphocyte apoptosis that has been well documented, as well as the roles of monocytes/macrophages cited above.

## Data Availability

Not applicable.
